# Serum asprosin level is significantly lower in patients with liver cirrhosis than in those with non-cirrhotic chronic liver diseases

**DOI:** 10.3389/fendo.2025.1691224

**Published:** 2025-12-12

**Authors:** Sichao Wang, Yan Wang, Shujun Zhang, Tao Li, Xinli Zhou, Li Chen

**Affiliations:** 1 Department of Infectious Disease and Hepatology, The Second Qilu Hospital of Shandong University, Jinan, China; 2 Department of Clinical Laboratory, Qilu Hospital of Shandong University, Jinan, China; 3 Department of Endocrinology, Shandong Provincial Hospital, Shandong University, Jinan, China; 4 Key Laboratory of Endocrine Glucose and Lipids Metabolism and Brain Aging, Ministry of Education, Jinan, China; 5 Department of Endocrinology and Metabolism, Qilu Hospital of Shandong University, Jinan, China; 6 Shandong Provincial Key Laboratory of Spatiotemporal Regulation and Precision Intervention in Endocrine and Metabolic Diseases, Jinan, China; 7 Shandong Provincial Engineering Research Center for Advanced Technologies in Prevention and Treatment of Chronic Metabolic Diseases, Jinan, China; 8 Institute of Endocrine and Metabolic Diseases of Shandong University, Jinan, China

**Keywords:** asprosin, adipokine, fibrosis-4 index, liver stiffness measurement, liver cirrhosis

## Abstract

**Aims:**

Given the paucity of research on the role of asprosin in liver fibrosis secondary to diverse chronic liver diseases, this study aimed to elucidate the association between serum asprosin levels and the development of liver cirrhosis.

**Methods:**

A total of 268 patients with diverse chronic liver diseases and 100 sex-matched healthy controls were enrolled. Participants were stratified into three groups: healthy controls (n=100), patients with non-cirrhotic chronic liver disease (n=62), and patients with liver cirrhosis (n=206). Serum asprosin levels were quantified using enzyme-linked immunosorbent assay. Additionally, all participants underwent assessments of anthropometric parameters, biochemical markers, and liver transient elastography.

**Results:**

Serum asprosin levels were significantly lower in patients with liver cirrhosis compared to those with non-cirrhotic chronic liver disease (p < 0.01) and healthy controls (p < 0.001). In patients with chronic liver disease, asprosin levels exhibited a negative correlation with the liver fibrosis markers Fibrosis-4 Index (r = -0.309, p < 0.001) and Liver Stiffness Measurement (r = -0.225, p = 0.006). Binary logistic regression analysis, after adjusting for multiple confounding factors (such as age, sex and body mass index, etc.), confirmed lower asprosin levels were independently associated with a higher risk of cirrhosis (all OR < 1, p < 0.05). Receiver Operating Characteristic curve analysis revealed that asprosin exhibited limited but statistically significant discriminative ability in differentiating between non-cirrhotic chronic liver disease and liver cirrhosis (AUC = 0.627, P = 0.002).

**Conclusions:**

These findings suggest that asprosin may be involved in the progression of liver cirrhosis, with potential as a novel biomarker for its diagnosis and prognosis and implications for fibrosis management. However, further prospective studies are needed to clarify the causal relationship between asprosin and liver cirrhosis and explore its underlying molecular mechanisms.

## Introduction

Chronic liver diseases (CLDs) constitute a major global burden of morbidity and mortality, with primary etiologies encompassing chronic hepatitis B virus and hepatitis C virus infections, alcoholic liver disease (ALD), metabolic dysfunction-associated steatotic liver disease (MASLD), autoimmune liver diseases, and other causes (1). Irrespective of the underlying cause, all CLDs follow a common histopathological trajectory characterized by hepatic fibrosis—the progressive accumulation of fibrous tissue that disrupts liver architecture, ultimately culminating in cirrhosis, the defining feature of advanced disease (2). Cirrhosis can lead to many severe complications, including variceal bleeding, ascites, spontaneous bacterial peritonitis, hepatic encephalopathy, hepatocellular carcinoma (HCC), and even death (3). Critically, fibrosis is reversible with early intervention, and timely diagnosis coupled with standardized management can slow or even reverse disease progression—this underscores its clinical importance in guiding pivotal decisions, such as reinforcing behavioral modifications in ALD or MASLD, determining indications for antiviral therapy in chronic viral hepatitis, and monitoring for complications and HCC (4). While liver biopsy remains the gold standard for histologic staging and grading of fibrosis, its invasiveness, inherent risk of complications, and impracticality for large-scale screening amid the high prevalence of CLDs necessitate alternative non-invasive approaches (5). These include serum-based fibrosis markers (e.g., hyaluronic acid, laminin), transient elastography (e.g., FibroScan), liver ultrasonography, and magnetic resonance imaging. However, each of these non-invasive modalities currently has inherent limitations in clinical diagnostics (6). Consequently, there is an urgent need to identify novel fibrosis-related biomarkers that can complement existing non-invasive methods to improve diagnostic accuracy and potentially serve as therapeutic targets for mitigating or reversing liver fibrosis.

Adipose tissue, once considered merely a passive reservoir for energy storage, has been redefined as one of the body’s most vital active endocrine and paracrine organs. It is responsible for synthesizing and secreting a diverse spectrum of adipokines, which play a pivotal role in regulating numerous physiological processes (7). As a newly identified adipokine, asprosin is primarily secreted by white adipose tissue and characterized by unique biological functions. As reported by Romere et al. (8), this 140-amino-acid protein is derived from the C-terminal cleavage of profibrillin—a precursor protein encoded by the FBN1 gene. In-depth mechanistic investigations have demonstrated that asprosin can cross the blood-brain barrier, enabling its interaction with hypothalamic neurons. This interaction promotes the secretion of agouti-related peptide—a key regulator of appetite—via the Gαs-cAMP-PKA signaling cascade, ultimately increasing appetite and potentially contributing to significant weight gain and obesity (9). In the liver, asprosin binds to the olfactory receptor Olfactory Receptor 734 in a concentration-dependent and saturable manner, activating the G protein-cAMP-PKA pathway to enhance hepatic glucose production, thereby elevating serum glucose and insulin levels (10). Furthermore, several animal studies have demonstrated that asprosin can effectively inhibit myocardial fibrosis and ameliorate myocardial injury (11, 12). While previous basic and clinical studies have established a close association between asprosin and non-alcoholic fatty liver disease (NAFLD) (13, 14), its role in other common types of chronic liver diseases remains to be elucidated—particularly in the context of cirrhosis, for which relevant research is currently lacking. Given asprosin’s significant role across multiple organs—with the liver as a key target organ—there is a critical need to investigate its concentrations in patients with common CLDs and cirrhosis. Such investigations are essential to determine whether asprosin levels change at the onset of hepatic fibrotic lesions, as this understanding could facilitate early diagnosis of CLDs via asprosin, potentially preventing progression to liver fibrosis, cirrhosis, and end-stage liver disease. Prior studies have focused on the association between asprosin and NAFLD/MASLD. However, the role of asprosin in other common etiologies of chronic liver disease (e.g., viral hepatitis, alcoholic liver disease, autoimmune liver disease) and its correlation with objective fibrosis markers (Fib-4 index and liver stiffness measurement) remain unexplored. Thus, in this study, we recruited participants with various CLDs, cirrhosis secondary to these CLDs, and healthy controls to compare asprosin levels across the three groups, and further explored the relationships between asprosin and markers of liver fibrosis as well as various metabolic parameters. This investigation aims to identify a novel perspective for the diagnosis and management of cirrhosis developing on the basis of multiple chronic liver diseases.

## Materials and methods

### Study participants

From October 2022 to July 2025, 268 patients with chronic liver disease of diverse etiologies were enrolled from the Department of Hepatology Ward at The Second Qilu Hospital of Shandong University. Among these patients, 206 cases who met the diagnostic criteria for liver cirrhosis (15) were assigned to the liver cirrhosis group, and the remaining 62 without cirrhosis were classified into the non-cirrhotic chronic liver disease group. During the same period, 100 healthy individuals who underwent physical examinations at the Health Check-up Center of the hospital were included as the healthy control group. All patients with chronic liver disease met the corresponding diagnostic and treatment criteria, expert consensus, or clinical guidelines, such as those for chronic hepatitis B and ALD (16, 17), as well as various other chronic liver diseases (18–21). Among the study participants, there were 223 male and 145 female patients, with a mean age of 55.51 ± 11.26 years. The inclusion criteria for the healthy control group were as follows: (1) aged > 18 years; (2) no history of abnormal liver ultrasound findings or liver function test results; and (3) normal liver ultrasound imaging and liver function test results at present. The exclusion criteria for the chronic liver disease population were as follows: (1) co-infection with human immunodeficiency virus; (2) acute cardiovascular diseases; (3) acute renal failure, severe renal insufficiency, or undergoing dialysis; (4) a history of liver transplantation; (5) acute hepatitis; (6) immunodeficiency states; (7) malignant tumors of other organs; (8) pregnant or breastfeeding women.

The study was approved by the Ethics Committee of The Second Qilu Hospital of Shandong University. Strict adherence to the principles of the Declaration of Helsinki was maintained to ensure the protection of human rights throughout the research process.

### Anthropometric data collection

Weight and height of all participants were measured and recorded by trained nurses. Body mass index (BMI) was calculated as weight in kilograms divided by the square of height in meters (kg/m²). For blood pressure measurement, participants were instructed to rest quietly for at least 10 minutes prior to testing. Measurements were performed three times for each participant, and the average of these readings was taken as the final result.

### Biochemical assessments

After a minimum of 8h of overnight fasting, blood specimens were collected from participants between 07:30 and 08:30 a.m. and immediately centrifuged to separate serum and cellular components. In the hospital’s clinical laboratory, following standardized procedures, serum levels of liver function markers including alanine aminotransferase (ALT, U/L), aspartate aminotransferase (AST, U/L), alkaline phosphatase (ALP, U/L), gamma-glutamyl transferase (GGT, U/L), total bilirubin (TBIL, μmol/L), direct bilirubin (DBIL, μmol/L), indirect bilirubin (IBIL, μmol/L), total protein (TP, g/L), albumin (ALB, g/L), and globulin (GLO, g/L); renal function markers such as creatinine (Cr, μmol/L), blood urea nitrogen (Urea, mmol/L), estimated glomerular filtration rate (eGFR, mL/min/1.73m²), and uric acid (UA, μmol/L); serum lipid parameters comprising triglycerides (TG, mmol/L), total cholesterol (TCH, mmol/L), low-density lipoprotein cholesterol (LDL-C, mmol/L), and high-density lipoprotein cholesterol (HDL-C, mmol/L); as well as fasting blood glucose (FBG, mmol/L), complete blood count indicators [white blood cells (WBC, ×10^9^/L), red blood cells (RBC, ×10¹²/L), hemoglobin (Hb, g/L), platelets (PLT, ×10^9^/L)], and alpha-fetoprotein (AFP, ng/mL) were measured. Remaining fasting serum samples were preserved at −80°C for subsequent asprosin analysis. The Fibrosis-4 index (Fib-4), which reflects the degree of liver fibrosis, was calculated using the formula: [Age (years) × AST (U/L)]/[PLT (×10^9^/L) × (ALT (U/L))^(1/2)] (22). The triglyceride-glucose (TyG) index, which quantifies insulin resistance by integrating fasting glucose and triglyceride levels, was calculated using the formula: TyG = ln[TG (mg/dL) × FBG (mg/dL)/2] (23).

### Measurement of serum asprosin

Venous blood samples were collected the following morning after an 8–10 hour fast, followed by centrifugation at 3000 rpm for 15 minutes to separate serum. Serum asprosin concentrations were quantified using an enzyme-linked immunosorbent assay kit (Shanghai Jianglai Industrial Co., Ltd.). All serum samples were stored at -80°C. Strict adherence to the manufacturer’s protocols for kits and instruments was maintained throughout the process. To ensure consistency, all participant samples underwent identical processing procedures and were subjected to the same number of freeze-thaw cycles. For enhanced accuracy, each sample was analyzed in duplicate and processed in a random order.

### Measurement of liver stiffness and controlled attenuation parameter

Liver stiffness (LSM) and controlled attenuation parameter (CAP) were quantified using FibroScan (Echosens, Paris, France). Participants were placed in a supine position with their right arm raised to expose the intercostal spaces over the right hepatic lobe. The probe was positioned perpendicular to the skin within the intercostal space for measurements. A total of 10 valid measurements were obtained for each participant, with accuracy verified for all successful acquisitions. The final result for both parameters was determined as the median of these 10 valid measurements. CAP was expressed in decibels per meter (dB/m), and LSM in kilopascals (kPa).

### Statistical analysis

Statistical analyses were performed using SPSS version 26 (IBM Corp, Armonk, NY, USA) and GraphPad Prism version 9 (GraphPad Software, Inc., San Diego, CA, USA). To determine the distribution characteristics of the dataset, the Kolmogorov–Smirnov test and P-P plots were employed. For normally distributed datasets, data are presented as means ± standard deviations. For non-normally distributed datasets, data are presented as medians with interquartile ranges (IQRs, 25th-75th percentiles). The chi-square test was used to compare categorical variables among different groups. For comparisons across multiple groups, one-way analysis of variance (ANOVA) or the Kruskal–Wallis test was chosen according to the data distribution pattern, and the Bonferroni correction method was applied for *post-hoc* analysis. Spearman analysis was used to assess the associations of serum asprosin level with various factors. Variables with statistically significant correlations were incorporated into the multiple linear regression model for analysis. Multiple linear regression analysis was performed to identify variables associated with serum asprosin levels in the population with liver diseases. To mitigate the influence of potential confounders on liver cirrhosis risk assessment, binary logistic regression models were employed to estimate the odds ratios (ORs) and their 95% confidence intervals (CIs). Model 1 (crude) included only asprosin as the independent variable. Model 2 adjusted for age and sex, the most basic confounding factors. Model 3 further adjusted for anthropometric parameters (height, weight, SBP, DBP, BMI) related to metabolic status. Model 4 added liver function markers (ALT, AST, ALB, GGT, TBIL) to account for liver injury. Model 5 included renal function indices (Cr, Urea, eGFR), and Model 6 incorporated metabolic parameters (FBG, TG, TC, LDL-C, HDL-C) to control for systemic metabolic disturbances. In these models, the dependent variable was liver cirrhosis, coded as 1 (present) versus 0 (absent). Receiver operating characteristic (ROC) curve analysis was performed to explore the potential diagnostic value of asprosin in distinguishing liver cirrhosis from chronic liver diseases. A p-value of less than 0.05 was considered statistically significant.

## Results

### Characteristics of participants across different groups

A total of 268 patients with chronic liver diseases of diverse etiologies were enrolled, including 150 cases of chronic hepatitis B, 11 cases of chronic hepatitis C, 52 cases of ALD, 12 cases of autoimmune hepatitis, 21 cases of primary biliary cholangitis, 6 cases of MASLD, and 16 cases of chronic liver diseases of unknown etiology. Among them, 206 patients (76.87%) had liver cirrhosis. Meanwhile, 100 healthy individuals were enrolled.

**Figure 1:**
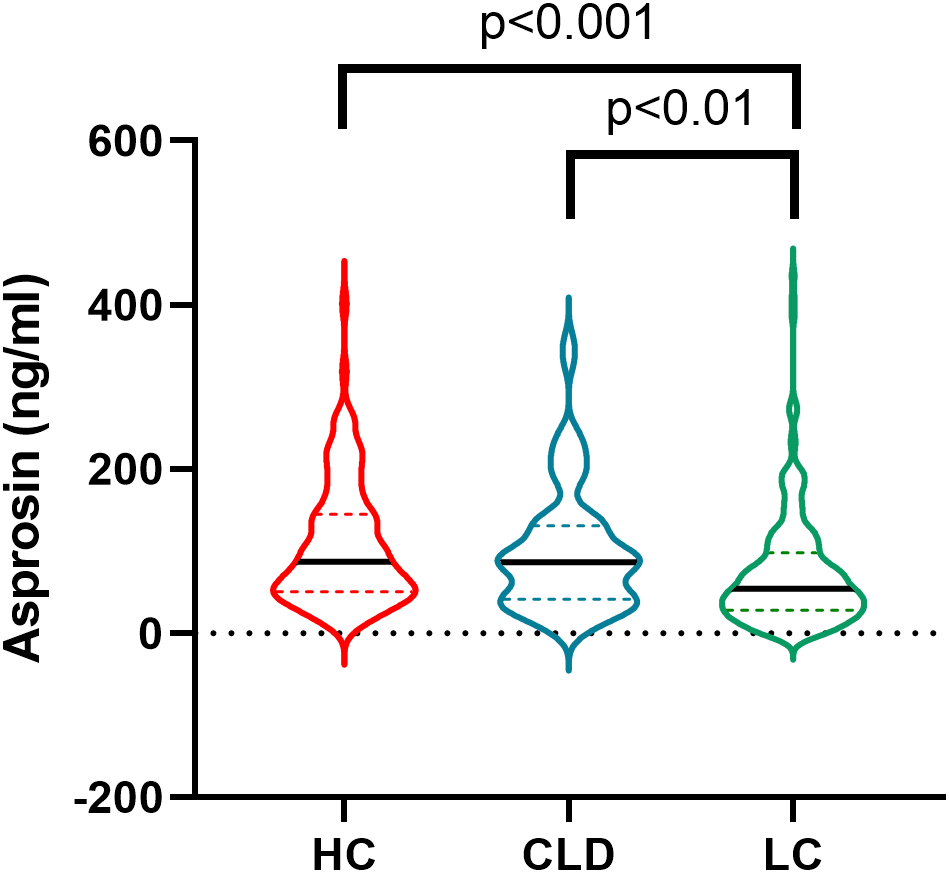
Circulating level of asprosin in each group. HC, healthy controls; CLD, patients with non-cirrhotic chronic liver disease; LC, patients with liver cirrhosis.

**Table 1** summarizes the clinical and biochemical characteristics of 100 healthy controls (HC), 62 patients with non-cirrhotic chronic liver disease (CLD), and 206 patients with liver cirrhosis (LC). Serum asprosin levels showed no significant difference between HC and CLD (68.46 ng/ml vs 83.67 ng/ml, p > 0.05), but differed significantly in LC (54.32 ng/ml) compared with both groups (p < 0.001 vs HC; p < 0.01 vs CLD) (**Figure 1**). Compared with HC and CLD, the LC group had older age and lower BMI. Regarding liver function, ALT, AST, ALP, and GGT showed distinct group differences across the three groups. Bilirubin parameters (TBIL, DBIL, IBIL) were lower in CLD than in HC but significantly higher in LC than in both groups. ALB levels also differed significantly across groups. Renal function indices (urea and eGFR) and lipid profiles (TG, TCH, LDL-C, HDL-C) varied significantly among the three groups. Hematological parameters, including WBC, RBC, Hb, and PLT, were lower in LC compared with HC and CLD. FBG and TyG showed significant intergroup differences, with FBG being relatively higher in the LC group. AFP was significantly lower in HC compared with the other two groups. The Fib-4 was significantly elevated in LC compared with the other two groups. Additionally, LSM was significantly higher in LC than in CLD, whereas CAP was significantly lower in LC versus CLD.

**Table 1:** Comparison of the clinical characteristics of patients between HC, patients with CLD, and patients with LC.

Characteristics	HC (n = 100)	CLD (n = 62)	LC (n = 206)	P
Age (years)	53.40 ± 7.79	49.47 ± 11.56	58.35 ± 11.68 a**,b***	<0.001
Sex (male/female)	56/44	28/34	139/67 b**	0.004
BMI (kg/m²)	25.75 ± 3.36	25.87 ± 3.94	24.45 ± 3.84 a*,b*	0.007
SBP (mmHg)	136.53 ± 17.61	132.93 ± 17.88	134.49 ± 18.14	0.471
DBP (mmHg)	88.76 ± 12.00	85.04 ± 11.91	82.12 ± 11.84 a***	<0.001
ALT (U/L)	21.40(14.00,32.75)	36.50(19.00,96.25) a***	22.00(14.00,36.00) a*,b**	<0.001
AST (U/L)	28.00(20.00,52.00)	41.00(22.75,89.25) a***	38.00(25.00,61.00) a***	<0.001
ALP (U/L)	97.00(73.00.140.00)	94.00(70.50,134.25) a***	109.00(82.00,156.00) a***	<0.001
GGT (U/L)	38.00(18.00,108.00)	74.50(26.75,147.00) a***	53.00(24.75,130.50) a***	<0.001
TBIL (μmol/L)	20.00(11.40,46.30)	14.10(8.78,20.90) a*	31.50(17.95,72.38) a***,b***	<0.001
DBIL (μmol/L)	10.40(4.55,28.55)	6.65(4.25,14.55) a***	16.45(8.93,45.33) a***,b***	<0.001
IBIL (μmol/L)	8.90(5.60,15.80)	6.05(4.08,9.45)	11.40(7.00,22.13) a***,b***	<0.001
TP (g/L)	70.94 ± 3.06	68.13 ± 7.16 a*	62.83 ± 8.32 a***,b***	<0.001
ALB (g/L)	36.75(30.90,43.92)	41.40(38.75,44.25) a***	32.75(28.70,36.73) a***,b***	<0.001
GLO (g/L)	27.30(23.40,33.10)	26.65(23.38,31.13)	29.25(23.45,34.80) a**	0.001
Cr (μmol/L)	62.00(53.00,73.00)	58.00(50.75,68.25)	62.00(53.00,75.00)	0.155
Urea (mmol/L)	4.90(3.80,5.90)	3.90(3.05,5.23) a*	5.04(3.90,6.50) b***	<0.001
eGFR (mL/min/1.73m²)	106.16 ± 12.53	116.29 ± 19.79 a*	110.64 ± 36.11	0.044
UA (μmol/L)	276.00(220.00,330.70)	266.00(215.50,345.75)	263.00(194.00,339.00)	0.242
TG (mmol/L)	0.95(0.70,1.34)	1.16(0.93,1.63) a**	0.91(0.64,1.24) b**	0.001
TCH (mmol/L)	4.85 ± 0.66	4.41 ± 1.37 a**	3.79 ± 1.44 a***,b**	<0.001
LDL-C (mmol/L)	2.69 ± 0.57	2.58 ± 1.10	2.03 ± 1.02 a***,b**	<0.001
HDL-C (mmol/L)	1.19(0.89,1.43)	1.14(0.85,1.42) a**	1.04(0.68,1.37) a***	<0.001
FBG (mmol/L)	5.14(4.71,5.95)	5.21(4.73,6.23) a*	5.32(4.73,6.80) a***	<0.001
TyG	8.22 ± 0.47	8.62 ± 0.65 a**	8.35 ± 0.62 b*	0.004
WBC (×10^9^/L)	5.00(3.55,6.49)	5.01(4.24,6.22) a**	4.08(3.11,6.00) a***,b*	<0.001
RBC (10¹²/L)	4.06(3.34,4.70)	3.45(4.00,4.88)	3.50(2.98,3.99) a***,b***	<0.001
Hb(g/L)	127.00(103.75,144.00)	140.50(124.00,149.75)	112.00(90.00,127.00) a***,b***	<0.001
PLT (×10^9^/L)	153.00(78.50,229.25)	200.00(141.25,255.75) a**	90.00(59.50,132.00) a***,b***	<0.001
AFP (ng/mL)	3.56(2.18,6.39)	4.15(2.13,8.72) a**	4.03(2.47,9.22) a***	<0.001
Fib-4	2.71(1.14,7.00)	1.57(1.01,3.69) a***	5.95(3.20,9.81) a***,b***	<0.001
Asprosin (ng/ml)	68.46(36.94,117.43)	86.36(41.37,131.12)	54.32(28.04,98.20) a***,b**	<0.001
LSM (kPa)	–	9.20(5.60,15.40)	17.35(10.53,30.80) b***	<0.001
CAP (dB/m)	–	253.60 ± 57.08	214.40 ± 58.56 b***	<0.001

HC, healthy controls; CLD, patients with non-cirrhotic chronic liver disease; LC, patients with liver cirrhosis. a: vs. HC, b: vs. CLD, *p < 0.05, **p < 0.01, ***p < 0.001.

BMI, Body Mass Index; SBP, Systolic Blood Pressure; DBP, Diastolic Blood Pressure; ALT, Alanine Aminotransferase; AST, Aspartate Aminotransferase; ALP, Alkaline Phosphatase; GGT, Gamma-Glutamyl Transferase; TBIL, Total Bilirubin; DBIL, Direct Bilirubin; IBIL, Indirect Bilirubin; TP, Total Protein; ALB, Albumin; GLO, Globulin; Cr, Creatinine; eGFR, Estimated Glomerular Filtration Rate; UA, Uric Acid; TG, Triglycerides; TCH, Total Cholesterol; LDL-C, Low-Density Lipoprotein Cholesterol; HDL-C, High-Density Lipoprotein Cholesterol; FBG, Fasting Blood Glucose; TyG, Triglyceride-Glucose Index; WBC, White Blood Cell Count; RBC, Red Blood Cell Count; Hb, Hemoglobin; PLT, Platelet Count; AFP, Alpha-Fetoprotein; Fib-4, Fibrosis-4 Index; LSM, Liver Stiffness Measurement; CAP, Controlled Attenuation Parameter.

### Correlation between serum asprosin levels and other indicators

**Figure 2:**
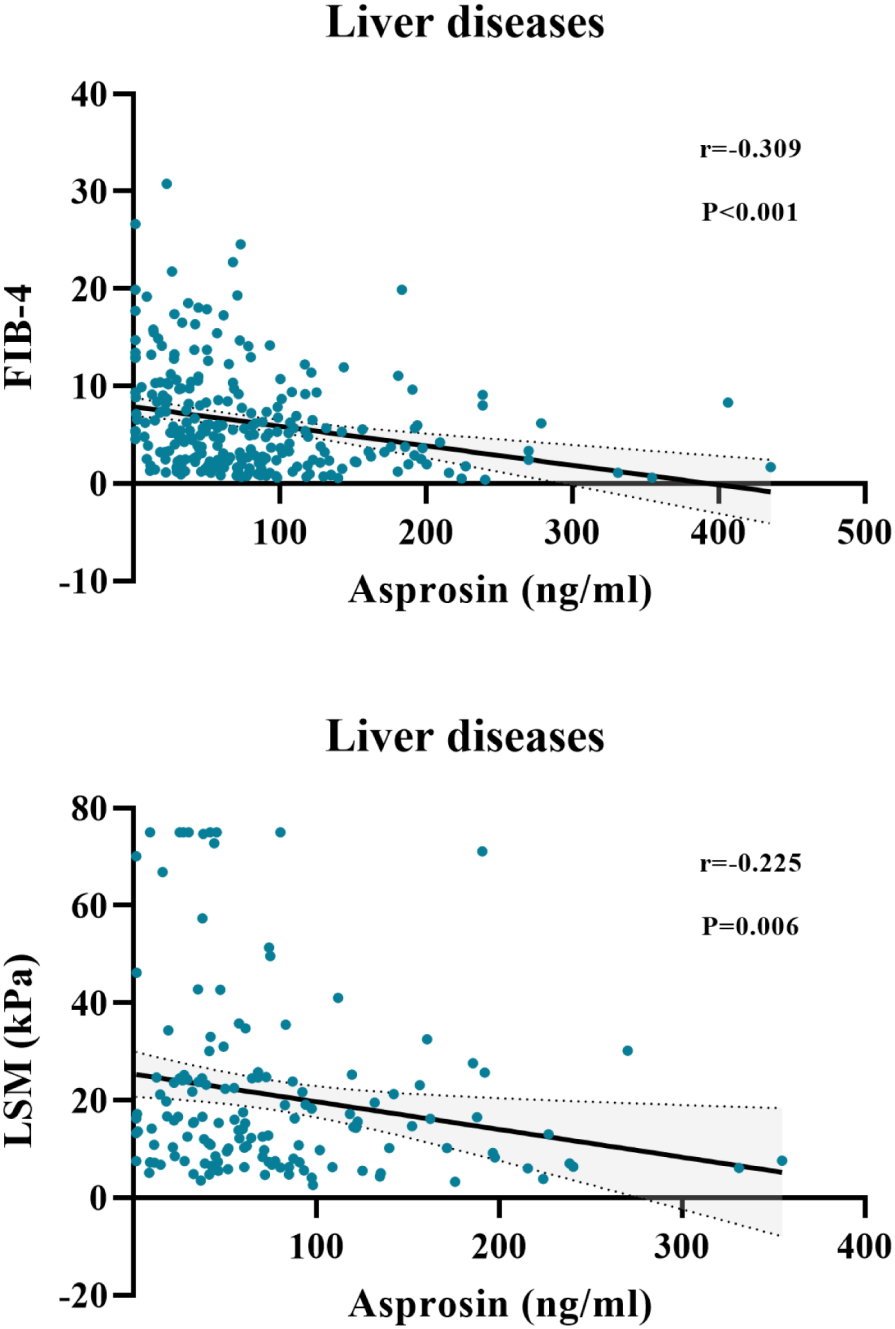
Scatterplots showing correlations of serum asprosin levels with Fib-4 and LSM in patients with liver diseases (CLD and LC).

In healthy controls (n = 100), serum asprosin showed no significant correlations with all analyzed indicators (all p ≥ 0.05). In patients with chronic liver diseases (n = 268), serum asprosin was positively correlated with ALP (r = 0.153, p = 0.012), GGT (r = 0.135, p = 0.027), Cr (r = 0.123, p = 0.049), TG (r = 0.174, p = 0.011), TCH (r = 0.134, p = 0.049), LDL-C (r = 0.172, p = 0.015), WBC (r = 0.415, p < 0.001), RBC (r = 0.166, p = 0.007), Hb (r = 0.122, p = 0.048), and PLT (r = 0.501, p < 0.001). Conversely, it was negatively correlated with Fib-4 (r = -0.309, p < 0.001) and LSM (r = -0.225, p = 0.006) (**Figure 2**). In the overall cohort (n = 368), serum asprosin was positively correlated with DBP (r = 0.111, p = 0.037), ALB (r = 0.136, p = 0.013), TCH (r = 0.149, p = 0.008), LDL-C (r = 0.156, p = 0.007), WBC (r = 0.380, p < 0.001), RBC (r = 0.244, p < 0.001), Hb (r = 0.190, p < 0.001), and PLT (r = 0.447, p < 0.001). Conversely, it was negatively correlated with TBIL (r = -0.125, p = 0.024), DBIL (r = -0.123, p = 0.026), and Fib-4 (r = -0.324, p < 0.001) (**Table 2**).

**Table 2:** Correlation analysis of asprosin with different indicators.

Indicators	Normal (n = 100)		Liver diseases (n = 268)		Overall (n = 368)	
	r	p	r	p	r	p
Age (years)	0.006	0.952	-0.004	0.949	-0.026	0.614
Sex (male/female)	0.027	0.793	-0.034	0.584	-0.008	0.877
BMI (kg/m²)	-0.141	0.182	0.058	0.386	0.038	0.502
SBP (mmHg)	-0.050	0.638	0.022	0.726	0.015	0.776
DBP (mmHg)	-0.035	0.744	0.102	0.099	0.111	0.037*
ALT (U/L)	0.043	0.670	0.019	0.760	-0.022	0.675
AST (U/L)	0.117	0.248	0.045	0.459	-0.090	0.084
ALP (U/L)	0.212	0.107	0.153	0.012*	0.094	0.090
GGT (U/L)	0.085	0.401	0.135	0.027*	0.010	0.842
TBIL (μmol/L)	-0.091	0.495	-0.069	0.258	-0.125	0.024*
DBIL (μmol/L)	-0.012	0.928	-0.063	0.308	-0.123	0.026*
IBIL (μmol/L)	-0.104	0.434	-0.075	0.224	-0.100	0.073
TP (g/L)	0.131	0.321	0.056	0.364	0.106	0.056
ALB (g/L)	0.141	0.260	0.049	0.420	0.136	0.013*
GLO (g/L)	0.038	0.776	-0.007	0.909	-0.026	0.635
Cr (μmol/L)	-0.072	0.479	0.123	0.049*	0.089	0.094
Urea (mmol/L)	-0.069	0.496	0.092	0.149	0.057	0.287
eGFR (mL/min/1.73m²)	0.037	0.731	-0.114	0.072	-0.105	0.053
UA (μmol/L)	-0.099	0.326	-0.035	0.624	-0.022	0.708
TG (mmol/L)	-0.133	0.188	0.174	0.011*	0.073	0.195
TCH (mmol/L)	-0.130	0.197	0.134	0.049*	0.149	0.008**
LDL-C (mmol/L)	-0.152	0.134	0.172	0.015*	0.156	0.007**
HDL-C (mmol/L)	0.120	0.237	-0.113	0.113	0.006	0.913
FBG (mmol/L)	0.134	0.184	-0.005	0.942	-0.023	0.670
TyG	-0.106	0.294	0.128	0.067	0.035	0.544
WBC (×10^9^/L)	0.011	0.914	0.415	<0.001***	0.380	<0.001***
RBC (10¹²/L)	0.040	0.693	0.166	0.007**	0.244	<0.001***
Hb (g/L)	0.047	0.642	0.122	0.048*	0.190	<0.001***
PLT (×10^9^/L)	0.024	0.816	0.501	<0.001***	0.447	<0.001***
AFP (ng/mL)	-0.074	0.486	-0.016	0.807	-0.096	0.081
Fib-4	0.032	0.749	-0.309	<0.001***	-0.324	<0.001***
LSM (kPa)	–	–	-0.225	0.006**	–	–
CAP (dB/m)	–	–	0.095	0.253	–	–

*p < 0.05, **p < 0.01, ***p < 0.001. BMI, Body Mass Index; SBP, Systolic Blood Pressure; DBP, Diastolic Blood Pressure; ALT, Alanine Aminotransferase; AST, Aspartate Aminotransferase; ALP, Alkaline Phosphatase; GGT, Gamma-Glutamyl Transferase; TBIL, Total Bilirubin; DBIL, Direct Bilirubin; IBIL, Indirect Bilirubin; TP, Total Protein; ALB, Albumin; GLO, Globulin; Cr, Creatinine; eGFR, Estimated Glomerular Filtration Rate; UA, Uric Acid; TG, Triglycerides; TCH, Total Cholesterol; LDL-C, Low-Density Lipoprotein Cholesterol; HDL-C, High-Density Lipoprotein Cholesterol; FBG, Fasting Blood Glucose; TyG, Triglyceride-Glucose Index; WBC, White Blood Cell Count; RBC, Red Blood Cell Count; Hb, Hemoglobin; PLT, Platelet Count; AFP, Alpha-Fetoprotein; Fib-4, Fibrosis-4 Index; LSM, Liver Stiffness Measurement; CAP, Controlled Attenuation Parameter.

These indicators included in the analysis were derived from those with statistically significant correlations in the previous correlation analysis. Utilizing multiple stepwise linear regression analysis with serum asprosin as the dependent variable and relevant indicators as independent variables among the population with liver diseases, the study found that TG (β = 24.006, SE = 10.338, t = 2.322, p = 0.023), TCH (β = -24.809, SE = 12.253, t = -2.025, p = 0.046), and PLT (β = 0.405, SE = 0.108, t = 3.738, p < 0.001) were independent influencing factors for serum asprosin (**Table 3**).

**Table 3:** Multiple linear regression analysis of variables associated with serum asprosin among the population with liver diseases.

Variables	β	SE	t	p
DBP (mmHg)	0.690	0.508	1.357	0.178
ALP (U/L)	0.139	0.103	1.344	0.182
GGT (U/L)	-0.028	0.039	-0.729	0.468
TBIL (μmol/L)	0.568	0.649	0.877	0.383
DBIL (μmol/L)	-0.453	0.810	-0.559	0.577
ALB (g/L)	-1.964	1.313	-1.496	0.138
Cr (μmol/L)	-0.174	0.257	-0.677	0.500
TG (mmol/L)	24.006	10.338	2.322	0.023*
TCH (mmol/L)	-24.809	12.253	-2.025	0.046*
LDL-C (mmol/L)	19.008	14.849	1.280	0.204
WBC (×10^9^/L)	-1.574	3.369	-0.467	0.642
RBC (10¹²/L)	-0.026	16.114	-0.002	0.999
Hb (g/L)	0.153	0.460	0.332	0.741
PLT (×10^9^/L)	0.405	0.108	3.738	<0.001***
Fib-4	-0.671	1.507	-0.446	0.657
LSM (kPa)	-0.390	0.344	-1.134	0.260

*p < 0.05, **p < 0.01, ***p < 0.001. DBP, Diastolic Blood Pressure; ALP, Alkaline Phosphatase; GGT, Gamma-Glutamyl Transferase; TBIL, Total Bilirubin; DBIL, Direct Bilirubin; ALB, Albumin; Cr, Creatinine; TG, Triglycerides; TCH, Total Cholesterol; LDL-C, Low-Density Lipoprotein Cholesterol; WBC, White Blood Cell Count; RBC, Red Blood Cell Count; Hb, Hemoglobin; PLT, Platelet Count; Fib-4, Fibrosis-4 Index; LSM, Liver Stiffness Measurement.

### Correlation between serum asprosin levels and the risk of liver cirrhosis in patients with chronic liver disease

As shown in the logistic regression models, with asprosin as the independent variable and liver cirrhosis as the dependent variable, the unadjusted odds ratio (OR) was 0.995, showing a statistically significant difference. After adjusting for age and sex, the OR was 0.993. Even after further adjustment for age, sex, height, weight, SBP, DBP, and BMI, the OR remained 0.992. Additional adjustment for ALT, AST, ALB, GGT, TBIL, Cr, Urea, eGFR, FBG, TG, TC, LDL - C, and HDL - C neither attenuated the magnitude of the ORs for liver cirrhosis nor affected the statistical significance (all p < 0.05) (**Table 4**).

**Table 4:** ORs for association between serum asprosin levels with liver cirrhosis.

Models	B	Wald	P	OR	95%CI
Model 1	-0.005	5.863	0.015	0.995	0.992-0.999
Model 2	-0.007	10.067	0.002	0.993	0.989-0.997
Model 3	-0.008	8.988	0.003	0.992	0.987-0.997
Model 4	-0.013	10.057	0.002	0.987	0.980-0.995
Model 5	-0.016	7.917	0.005	0.985	0.974-0.995
Model 6	-0.029	8.125	0.004	0.972	0.953-0.991

Model 1: crude.

Model 2: adjusted for age and sex.

Model 3: adjusted Model 2 + height, weight, SBP, DBP and BMI.

Model 4: adjusted Model 3 + ALT, AST, ALB, GGT, TBIL and ALB.

Model 5: adjusted Model 4 + Cr, Urea and eGFR.

Model 6: adjusted Model 5 + FBG, TG, TC, LDL-C and HDL-C.

### ROC analysis of serum asprosin for identifying liver cirrhosis in patients with chronic liver disease

**Figure 3:**
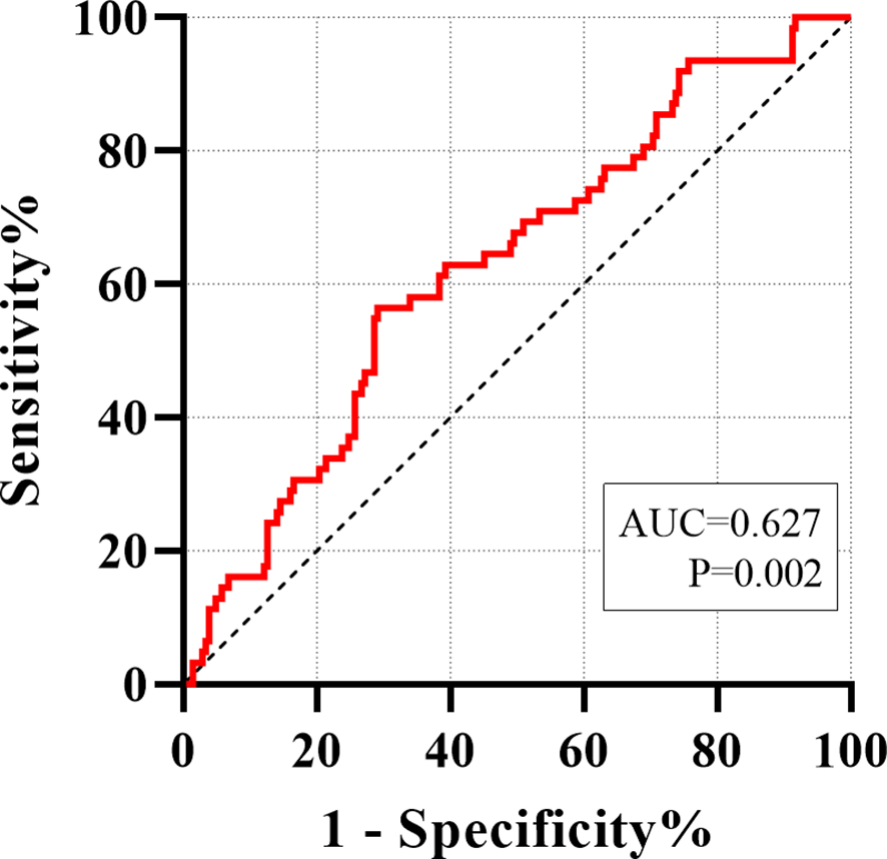
ROC analyze was carried out for distinguishing between non-cirrhotic chronic liver disease and liver cirrhosis.

The predictive value of serum asprosin for identifying liver cirrhosis in patients with chronic liver disease was evaluated using receiver ROC curve analysis. The area under the curve (AUC) was 0.627 (95% CI: 0.550–0.705, p = 0.002). At the optimal cutoff value of 82.55 ng/ml, the sensitivity and specificity were 56.45% and 70.87%, respectively (p < 0.001) (**Figure 3**).

## Discussion

This study employed a clinical cross-sectional design to quantify and analyze serum asprosin levels in healthy individuals, patients with non-cirrhotic chronic liver disease, and those with liver cirrhosis. The results demonstrated that serum asprosin levels were lower in patients with liver cirrhosis compared to those with non-cirrhotic chronic liver disease (p < 0.01) and healthy controls (p < 0.001), with statistically significant differences observed (**Table 1**, **Figure 1**). Among all patients with chronic liver disease, serum asprosin levels were significantly negatively correlated with the liver fibrosis indices Fib-4 (r = -0.309, p < 0.001) and LSM (r = -0.225, p = 0.006) (**Table 2**, **Figure 2**). Logistic regression analysis confirmed that asprosin had an independent yet mild protective association with liver cirrhosis after adjusting for multiple confounding variables, with all ORs and 95% CIs being < 1 (all p < 0.05) (**Table 4**). ROC curve analysis revealed that asprosin exhibited limited but statistically significant discriminative ability in differentiating between chronic liver disease and liver cirrhosis, with an AUC of 0.627 (p = 0.002) (**Figure 3**). These findings provide valuable insights into the role of asprosin in the development and progression of liver cirrhosis, and offer important evidence for exploring its potential clinical applications.

As an important adipokine, asprosin plays a key role in various physiological processes such as energy metabolism, immune regulation, and tissue repair (11, 24, 25). In recent years, its association with liver diseases has attracted increasing attention. Existing studies have found that asprosin expression is abnormal in liver diseases such as NAFLD, suggesting its potential involvement in hepatic pathophysiological processes (13, 26, 27). The result of significantly reduced serum asprosin levels in cirrhotic patients in this study provides direct clinical evidence for the close association between asprosin and liver cirrhosis.

As a key metabolic organ in the human body, the liver exhibits impaired capacity to process adipokines appropriately when its function is compromised (28), which may potentially result in disturbances in asprosin metabolism. Meanwhile, systemic inflammatory responses, oxidative stress, and visceral hemodynamic changes caused by portal hypertension in cirrhosis may inhibit the synthesis and secretion of asprosin by adipocytes (29, 30). Specifically, systemic inflammatory responses can activate immune cells, releasing a large number of inflammatory factors that directly act on adipocytes, interfering with the expression of genes related to asprosin synthesis. A large number of free radicals generated by oxidative stress damage organelles in adipocytes (31), affecting normal cellular physiological functions and thereby reducing asprosin synthesis efficiency. Visceral hemodynamic changes caused by portal hypertension reduce blood perfusion in adipose tissue, leading to insufficient supply of nutrients (32), which is also unfavorable for asprosin synthesis and secretion.

Notably, a prior study has demonstrated that asprosin can inhibit myocardial fibrosis by preserving the viability of mesenchymal stem cells (MSCs) (11), while accumulating evidence has further confirmed that MSCs exert a significant inhibitory effect on liver fibrosis (33, 34). This thus leads us to hypothesize that asprosin may potentially exert a therapeutic effect on liver fibrosis via MSCs, a proposition worthy of further investigation. MSCs, which possess strong self-renewal and multi-directional differentiation capabilities, can differentiate into hepatocyte-like cells within the hepatic microenvironment to replenish damaged hepatocytes and secrete various cytokines that regulate immune responses and anti-fibrotic processes. *In vitro* experiments have shown that asprosin inhibits hydrogen peroxide-induced oxidative stress and apoptosis in MSCs by activating the ERK1/2-SOD2 pathway (11). It is thus hypothesized that asprosin may inhibit hepatic fibrosis and delay the progression of cirrhosis by enhancing MSC activity and regulating the hepatic microenvironment. Another basic research, through *in vivo* and *in vitro* experiments, revealed that asprosin alleviates diabetes-induced myocardial fibrosis by suppressing excessive autophagy mediated by the AMPK/mTOR/ULK1 pathway (12). Based on prior studies, we hypothesize that asprosin may exert anti-fibrotic effects via potential mechanisms such as modulation of MSCs viability or inhibition of oxidative stress. However, these mechanisms are hypothesis-generating and have not been experimentally validated in this work. Future basic research, including *in vitro* studies on hepatic stellate cells and *in vivo* liver fibrosis models, is needed to elucidate the molecular mechanisms underlying the observed clinical association.

This study demonstrated that serum asprosin levels were significantly negatively correlated with Fib-4 and LSM across all included patients with liver disease, a finding that strongly supports the research hypothesis. Fib-4 is a classic score for evaluating liver fibrosis severity calculated from serological indicators, while LSM directly reflects liver stiffness through liver elastography; both can well reflect the severity of liver fibrosis (35). The negative correlation between asprosin and these two indicators indicates that asprosin is likely involved in the pathological progression of liver fibrosis, with its levels decreasing as liver fibrosis worsens. Existing studies have demonstrated that the activation of hepatic stellate cells constitutes a central link in the pathogenesis of liver fibrosis (36, 37), and asprosin may exert anti-fibrotic effects by inhibiting the activation, proliferation, and collagen synthesis of these cells. The results of correlation analysis of clinical data in this study further reinforce the protective role of asprosin in the progression of liver fibrosis.

Logistic regression analysis confirmed that asprosin had an independent yet mild protective association with liver cirrhosis, and this result remains valid after adjusting for confounding variables such as age, gender, blood pressure, height, weight, BMI, liver and kidney function, blood lipids, and blood glucose. This fully indicates that reduced asprosin levels have an independent association with the development of liver cirrhosis. It is noteworthy that malnutrition and metabolic disorders, which are frequently associated with cirrhotic patients (38), may still indirectly affect asprosin levels by reducing body fat mass. Malnutrition results in nutrient deficiency in adipocytes, impairing the supply of raw materials for asprosin synthesis; metabolic disorders, on the other hand, may alter metabolic pathways within adipocytes, thereby interfering with the synthesis, processing, and secretion of asprosin. This suggests that asprosin may not merely represent an accompanying phenomenon in the onset of liver cirrhosis, but also exert an active protective role in its development and progression, with changes in its levels potentially involved in the pathophysiological mechanisms of the disease. This thus identifies a novel potential target for the prevention and treatment of liver cirrhosis; in the future, modulation of asprosin levels or its signaling pathways may offer a means to delay or even prevent the progression of this disease.

ROC curve analysis revealed that asprosin exhibits limited but statistically significant discriminative ability in differentiating liver cirrhosis from chronic liver disease, underscoring its clinical potential as a diagnostic marker for liver cirrhosis. Currently, a variety of non-invasive indicators are employed in clinical practice for the diagnosis of liver cirrhosis, yet each has its inherent limitations. For instance, the Fib-4 index is susceptible to misclassification in patients with metabolic syndrome or inflammatory conditions, whereas LSM measurements are significantly influenced by factors such as ascites and obesity (39, 40). The discovery of the diagnostic value of asprosin as a new potential marker provides new ideas for the early diagnosis of liver cirrhosis. If asprosin can be combined with existing indicators, it is expected to further improve diagnostic accuracy and specificity, reduce the need for invasive liver biopsy, which has important clinical practical value.

Building on the aforementioned associations, and considering previous basic experiments demonstrating that exogenous asprosin administration alleviates myocardial fibrosis when applied to cardiomyocytes (11, 12), we may even postulate that its therapeutic potential as an exogenous agent merits further investigation in future research. It is hypothesized that the administration of an appropriate dose of exogenous asprosin may, to a certain extent, inhibit the progression of liver fibrosis in patients with various chronic liver diseases, reduce the incidence of cirrhosis, and thereby transform asprosin from a diagnostic marker into an effective anti-fibrotic therapeutic agent. If this hypothesis can be verified by basic or clinical trials, it will open up a new path for the treatment of liver fibrosis, and significantly improve patient prognosis.

This study also has certain limitations. First, given the cross-sectional nature of this study, we cannot establish a causal relationship between reduced asprosin levels and liver cirrhosis progression. The lower asprosin levels in cirrhotic patients may be a consequence of liver dysfunction or systemic metabolic disturbances associated with cirrhosis, rather than a direct driver of fibrosis. To verify the predictive value of asprosin, future longitudinal studies should enroll patients with early-stage chronic liver disease and dynamically monitor serum asprosin levels over 2–5 years. Correlating changes in asprosin with fibrosis progression (assessed via repeated LSM or Fib-4) and clinical outcomes will help clarify whether asprosin serves as a prognostic marker for disease advancement or regression. Second, this study did not investigate interactions between asprosin and other cytokines or inflammatory mediators, and the specific molecular mechanisms underlying its protective effects remain to be elucidated through further basic experimental research. Additionally, the sample size of this study is relatively limited, and subgroup analyses stratified by the etiology of cirrhosis were not performed, which may compromise the generalizability of the findings. The non-cirrhotic CLD group was relatively small compared to the cirrhosis group, which may have biased statistical comparisons. This imbalance could affect the generalizability of our findings, particularly regarding the differential diagnostic value of asprosin between these two groups. Finally, since the expression of asprosin in liver tissue was not assessed, its local role within the liver cannot be definitively established.

In conclusion, this study confirms that serum asprosin levels are significantly reduced in cirrhotic patients and are closely related to the degree of liver fibrosis. These findings suggest that asprosin may be associated with the progression of liver cirrhosis, with potential as a novel biomarker for its diagnosis and potential implications for fibrosis management. However, due to the cross-sectional design, this study cannot establish a causal relationship between asprosin and liver cirrhosis. Further prospective studies are needed to clarify the causal relationship and explore its underlying molecular mechanisms, as well as to verify the prognostic value of asprosin.

## Data Availability

The datasets generated and/or analyzed during the current study are not publicly available due to institutional restrictions but are available from the first author (Wang Sichao, 15315583419@163.com) upon reasonable request and with approval from the Ethics Committee of The Second Qilu Hospital of Shandong University. Requests to access the datasets should be directed to Wang Sichao, 15315583419@163.com.
